# Comment on: Legal but persecuted: The cultural politics of therapeutic abortion in Latin America

**DOI:** 10.1371/journal.pgph.0006448

**Published:** 2026-05-08

**Authors:** Silvana Matassini Eyzaguirre, Magaly Blas

**Affiliations:** 1 McGill School of Population and Global Health, McGill University, Montreal, Canada; 2 CINAPS, Centro de Investigación en Atención Primaria de Salud (CINAPS) de la Universidad Peruana Cayetano Heredia (UPCH), Lima, Perú; 3 Univesidad Peruana Cayetano Heredia (UPCH), Lima, Perú; PLOS: Public Library of Science, UNITED STATES OF AMERICA

The recognition and protection of women’s sexual and reproductive rights remain far from secure. Across the world, these rights continue to be contested terrain, shaped by political shifts, moral discourses, economic inequalities, and deeply entrenched gender hierarchies. While framed as universal human rights, access to sexual and reproductive healthcare is profoundly unequal. Across the Americas, regressive movements, emboldened by policies from the Trump administration, are dismantling decades of progress in sexual and reproductive health. Conservative forces continue to dictate how women’s bodies should be controlled, denied, or punished. In a world where autonomy remains politicized, the fight for therapeutic abortion is not only a medical issue but a profound struggle for justice, dignity, and freedom.

Ensuring safe, legal, and accessible therapeutic abortion is fundamental to advancing women’s health rights. Recent policies, such as the expanded Global Gag Rule [1] and cuts to international family planning aid, have had severe repercussions, particularly in Latin America, where many already face restrictive abortion laws and fragile healthcare systems. These measures have led to clinic closures, reduced contraceptive supplies, and limited access to safe abortion services, disproportionately affecting poor and rural women [2]. As Latin American activists warn, U.S. policy reversals risk setting back years of progress in gender equality and reproductive justice across the region [3].

Persistent poverty, entrenched socioeconomic inequality, and uneven access to comprehensive sexuality education and healthcare continue to shape how women and girls experience their bodies and reproductive lives. These structural conditions coexist with conservative currents that frame abortion, including its therapeutic form, legally permitted in several countries for decades, as morally suspect and politically contentious. Such tensions are reflected in a fragmented and shifting legal landscape: while some countries have expanded recognition of reproductive autonomy, others maintain highly restrictive frameworks.

Chile, traditionally restrictive, currently allows abortion only under three exceptions, risk to the mother’s life, fatal fetal malformation, and rape, while a new bill under congressional debate seeks to expand access by legalizing abortion on demand up to 14 weeks of gestation [4]. Argentina legalized abortion in 2020, allowing elective terminations up to 14 weeks, with later abortions permitted under limited conditions [5]. Peru maintains a more restrictive framework, permitting abortion only to save the life or health of the woman, in cases of rape or non-consensual insemination, or for severe fetal abnormalities [6]. Bolivia authorizes abortion under specific circumstances, including risk to the woman’s life, rape, incest, or when the woman is a minor, and the procedure generally requires judicial authorization [7]. Colombia is among the most progressive, decriminalizing abortion up to 24 weeks gestation [8]. Brazil and Uruguay allow abortion under Chile-like legal grounds, while Ecuador permits abortion in cases of risk to the life or health of the woman, or in pregnancies resulting from rape, including those involving minors and women with disabilities [9].

Advancement in this matter depends not only on legal reform but also on public education and health system improvements to ensure real access. However, conservative resistance remains strong across the region, shaping the practical access to these rights. In that sense, Peru exemplifies the challenges that remain. According to data from the Ministry of Women and Vulnerable Populations (MIMP) and based on annual records from the National Aurora Program [10], reports of sexual violence amount to approximately one case every 40 minutes. In addition, even though therapeutic abortion has been legal in Peru for over a century, permitted when the life or health of a pregnant person is at risk, this legal right has become increasingly difficult to exercise [9,11–13].

Between 2018 and 2023, police registered 79 complaints related to therapeutic abortion. Most hospitals, however, classify such cases as “unspecified” abortions, masking a widespread reluctance among clinicians to apply the law. This hesitation is driven largely by fear of persecution and accusations from “pro-family” groups supported by conservative right-wing sectors as well as segments of the radical left [9,11–13]. Women seeking emergency care are sometimes reported to prosecutors, facing long investigations even without a crime. This moralizing trend intensified in 2023, when Congress attempted to grant personhood to “the conceived” through a national fetal registry, restricting women’s sexual and reproductive rights. Although the law failed, it set a precedent that continues to shape power relations and influence medical practice [11].

Legislative efforts to restrict women’s access to this procedure continue unabated. Backed, again, by conservative parties, Peru’s revised Therapeutic Abortion Guidelines eliminated key clauses on mental health in cases of child rape and on congenital malformations incompatible with life. This change narrows the law’s clinical applicability by removing scenarios that once provided legal and medical safeguards. Their removal undermines guideline implementation, further endangering girls who are victims of sexual violence and distancing the State from its obligations on reproductive and human rights.

The recent case of a former Peruvian congresswoman [13] publicly asserting that pregnancies resulting from rape should be embraced as opportunities for motherhood, including in cases involving girls and adolescents, illustrates the moral politics at stake. Pregnancy was recast not as a continuation of violence against a child but as redemptive maternity. The primary victim was positioned as the “unborn child,” while the violated girl was displaced from moral concern. Protection was redirected away from minors subjected to sexual violence and toward fetal life, reordering the hierarchy of vulnerability.

While articulated by specific actors, such claims do not remain isolated opinions. From positions of institutional and symbolic authority, political and public figures advance moral arguments that gain traction through their amplification in mass media and political platforms. We describe this process as a form of *discursive authoritarianism*: a mode of power that does not initially rely on formal legal prohibition but instead operates through the normalization and amplification of moralizing claims. Public officials, leveraging privileged access to television, press, and digital media infrastructures, circulate conservative narratives that gradually sediment into taken-for-granted truths. Through repetition, spectacle, and affective appeals, these statements acquire normative force, delimiting what becomes socially intelligible as “justice,” “protection,” and “morality.”

By translating moral positions into administrative procedures, such measures reinforce institutional barriers, promote legal ambiguity, and normalize the surveillance of reproductive decisions within the health system. In Foucauldian terms [14], this reflects a biopolitical reordering of value: the state intensifies its protection of fetal life while disciplining and regulating the reproductive bodies of girls and women. Under these conditions, even legally recognized provisions, such as therapeutic abortion, risk becoming effectively inaccessible. Although formally permitted within the legal framework, the combination of moral pressure, procedural complexity, and institutional hesitation can delay, obstruct, or discourage its application in practice.

In the centennial year of its legal recognition, Peru’s therapeutic abortion law stands as both a historical milestone and a contemporary fiction. For global health and other countries worldwide, Peru’s experience exposes the gap between legality and legitimacy. This moral governance reproduces and institutionalises gender violence by turning the female body into both the site and symbol of moral restoration. Meanwhile, structural injustices, impunity for perpetrators, religious conservatism in public policy, and state neglect, remain unaddressed. Until these foundations are confronted, the promise of reproductive justice will persist as a legal fiction.
